# Dispersal similarly shapes both population genetics and community patterns in the marine realm

**DOI:** 10.1038/srep28730

**Published:** 2016-06-27

**Authors:** Guillem Chust, Ernesto Villarino, Anne Chenuil, Xabier Irigoien, Nihayet Bizsel, Antonio Bode, Cecilie Broms, Simon Claus, María L. Fernández de Puelles, Serena Fonda-Umani, Galice Hoarau, Maria G. Mazzocchi, Patricija Mozetič, Leen Vandepitte, Helena Veríssimo, Soultana Zervoudaki, Angel Borja

**Affiliations:** 1 AZTI, Herrera Kaia, Portualdea z/g—20110 Pasaia, Gipuzkoa, Spain; 2 IMBE, Aix Marseille Université, CNRS, IRD, Avignon Université, station marine d’Endoume, chemin de la Batterie-des-Lions, 13007 Marseille, France; 3 King Abdullah University of Science and Technology (KAUST), Red Sea Research Center (RSRC), Thuwal 23955-6900, Saudi Arabia; 4 IMST, Dokuz Eylul University, Baku Bulvarı No: 100, Izmir, Turkey; 5 Instituto Español de Oceanografía (IEO), Centro Oceanográfico de A Coruña, Apdo. 130, 15080 A Coruña, Spain; 6 Institute of Marine Research, Postboks 1870 Nordnes, 5817 Bergen, Norway; 7 Flanders Marine Institute—VLIZ, InnovOcean site, Wandelaarkaai 7, Oostende, Belgium; 8 Spanish Institute of Oceanography, Baleares Oceanographic Center, PO Box 291. 07015 Palma de Mallorca, Spain; 9 University of Trieste, Department of Biology, Via A. Valerio 28/A, 34127 Trieste, Italy; 10 University of Nordland, Faculty of Biosciences and Aquaculture, Bodø, Norway; 11 Stazione Zoologica Anton Dohrn, Villa Comunale, 80121 Napoli, Italy; 12 National Institute of Biology, Marine Biology Station, Fornace 41, 6330 Piran, Slovenia; 13 MARE (Marine and Environmental Sciences Centre), Faculdade de Ciências e Tecnologia, Universidade de Coimbra, 3004-517 Coimbra, Portugal; 14 Institute of Oceanography, Hellenic Centre for Marine Research, PO 712, 46.7 km Avenue Athens-Sounio, 19013 Anavyssos, Athens, Greece

## Abstract

Dispersal plays a key role to connect populations and, if limited, is one of the main processes to maintain and generate regional biodiversity. According to neutral theories of molecular evolution and biodiversity, dispersal limitation of propagules and population stochasticity are integral to shaping both genetic and community structure. We conducted a parallel analysis of biological connectivity at genetic and community levels in marine groups with different dispersal traits. We compiled large data sets of population genetic structure (98 benthic macroinvertebrate and 35 planktonic species) and biogeographic data (2193 benthic macroinvertebrate and 734 planktonic species). We estimated dispersal distances from population genetic data (i.e., F_ST_ vs. geographic distance) and from β-diversity at the community level. Dispersal distances ranked the biological groups in the same order at both genetic and community levels, as predicted by organism dispersal ability and seascape connectivity: macrozoobenthic species without dispersing larvae, followed by macrozoobenthic species with dispersing larvae and plankton (phyto- and zooplankton). This ranking order is associated with constraints to the movement of macrozoobenthos within the seabed compared with the pelagic habitat. We showed that dispersal limitation similarly determines the connectivity degree of communities and populations, supporting the predictions of neutral theories in marine biodiversity patterns.

Dispersal plays a key role to connect populations, and contrastingly, its moderate limitation is one of the main processes to maintain species coexistence and promote regional biodiversity^1^,^2^. Knowledge of population connectivity and dispersal is relevant for determining the resilience of species to global change^3^, the establishment of sustainable fisheries management strategies^4^, the design of networks of functional marine protected areas^4^,^5^,^6^, and other conservation issues, such as habitat restoration, population viability analysis, and invasive species monitoring^7^. However, difficulties associated with tracking and modelling the trajectory and fate of propagules and larvae have limited our knowledge of dispersal strategies and population connectivity of many marine species^8^.

Dispersal limitation of propagules and larvae and their demographic stochasticity (i.e. resulting from random events of individual mortality and reproduction, and not from environmental variance which can also induce population fluctuations) are neutral processes that shape both genetic structure and community composition. Due to finite number of individuals in a population or community, the relative frequencies of alleles or species will to some degree change stochastically^9^. Recently, studies have been motivated to identify similarities between processes underlying patterns of species diversity and those underlying genetic diversity^10^,^11^,^12^,^13^,^14^,^15^. In neutral theories, alternative forms of a gene (alleles or haplotypes) in a population are analogous to species in a community, random genetic drift in populations is analogous to ecological drift (random fluctuations in species relative abundances^16^) in communities, and spatially structured populations (i.e., metapopulations) are analogous to metacommunities^17^. The neutral theory of molecular evolution^18^ states that most evolutionary changes at the molecular level are the result of random genetic drift acting on neutral alleles (those that do not affect fitness). When the number of migrants that disperse over short distances is higher than that over long distances, the isolation-by-distance (IBD) theory predicts that pairwise genetic variation (for instance, the Wright’s fixation index F_ST_, the sample pairwise genetic differentiation) will increase with the geographic distance between a pair of populations^18^,^19^,^20^; see Fig. 1a. Quantitative IBD predictions consider neutral alleles and populations to be at equilibrium between dispersal and genetic drift^21^. The slope of IBD varies with migration rate (i.e., the proportion of individuals that leave the natal site and successfully reproduce at another site) (Fig. 1a) and is commonly used for estimating dispersal distance (i.e., geographic distance travelled between source and settlement sites) with genetic markers.

In ecology, whether the regional distribution of species arises from limitations to dispersal^22^ or niche adaptive processes^23^ has been a long-standing debate and the emergence of the concept of neutrality^16^ has appeared more recently than it has in population genetics. In a neutral community, all individuals are assumed to have the same prospects of reproduction and death. According to the neutral model of biodiversity, species cross-site similarity (i.e., the opposite of β-diversity) is predicted to decline logarithmically with increasing geographical distance when migration rate is low^15^,^16^ (see Fig. 1b). This pattern, named distance decay, has been observed for a variety of biomes and taxa: trees of the rainforest^24^,^25^, coral reefs^16^, marine bacteria^26^, and plankton^27^ (but see ref. 28). Neutral theories of macroecology have synthesised spatial patterns in species diversity and genetic diversity that postulate that stochastic processes (migration, genetic/ecological drift, and mutation/speciation) act similarly at all taxonomic scales down to the level of individuals^11^. However, parallels in biological connectivity between population genetics and community ecology have been nearly exclusively restricted to theoretical studies^12^,^29^ that have been validated with field observations in only a few terrestrial and freshwater groups^10^,^11^,^13^,^17^,^30^,^31^; none of which represent the marine realm.

Our aim is to evaluate whether dispersal traits in marine species determine the connectivity degree among communities and among populations within species. In particular, we hypothesise that planktonic species will have a higher dispersal distance than macrozoobenthic species at both the genetic and community levels. We base this prediction on constraints to movement in adult macroinvertebrates within the seabed, which are only partially compensated for by their larval stage. In comparison, pelagic plankton experience higher seascape connectivity. To test this hypothesis, first, we conducted a meta-analysis based on a literature survey of the genetic population structure (98 macrozoobenthic species and 35 planktonic species) and collated a large data set on community composition (2193 macrozoobenthic species and 734 planktonic species). Subsequently, we estimated dispersal distances at the genetic level derived from IBD slopes (i.e., F_ST_ vs. geographic distance) and compared them with those at the community level derived from β-diversity analysis.

## Results

### Population genetic analysis

Our literature search for studies of population genetic analysis found 12 on phytoplankton (addressing 13 species), 42 on zooplankton (22 species), and 110 on macrozoobenthos (98 species) (Table S1). From these 98 macrozoobenthic species, 81 species have dispersing larvae (DL) and 17 species have nondispersing larvae (NDL); 62 species live on hard bottoms and 36 species live in mixed- or soft-sediment habitat.

**Figure 2:**
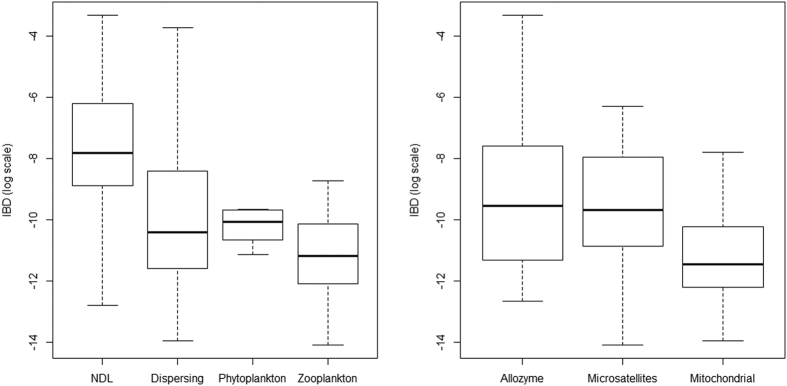
Boxplots of IBD slopes according to biological group or molecular marker type. NDL = macrozoobenthos Non-Dispersing Larvae, Dispersing: macrozoobenthos Dispersing Larvae.

The mean IBD slopes for each biological group or marker type are shown in Table 1 and Fig. 2. The two-way analysis of variance (ANOVA) indicated significantly different IBD slopes among groups for both factors (p < 0.0001 for the biological group, p = 0.009 for marker type, and p = 0.012 for their interaction using the logarithm of IBD slope to normalise distribution (Kolmogorov-Smirnov p = 0.194)). Biological group was the main factor explaining variance (17.4%, compared with 6.0% by marker type and 9.4% by interaction). A Tukey’s post-hoc test showed that significant differences exist between the IBD slopes of NDL and DL macrozoobenthic species, between those of zooplankton and DL macrozoobenthic species, and between those of zooplankton and NDL macrozoobenthic species (Table 2). These results indicate that IBD slope is significantly higher for NDL, moderate for DL and lower for zooplankton (Fig. 2). Potentially the small sample size of studies limited the differentiation of phytoplankton from any other group. However, when phytoplankton and zooplankton are clumped into a single group (i.e., plankton), its IBD slope was significantly larger than that of NDL (p < 0.0001) or DL (p = 0.035). Using the power function model established in Palumbi^32^ (see methods), we inferred dispersal scales for each biological group from their IBD slopes. The inferred dispersal scales were as follows: NDL macrozoobenthic species (0.31 km) <DL macrozoobenthic species (1.92 km) <phytoplanktonic species (19.5 km) < zooplanktonic species (88.9 km) (Table 1).

In the meta-analysis, which takes into account different weights assigned to the different studies, the test of moderators indicated significant differences among biological groups (QM_(df=5)_ =17.48, p = 0.0037); in particular, species of NDL had significantly higher logarithmic IBD slopes (p = 0.0004) compared with the overall mean. In contrast, no molecular marker type was significantly different in terms of logarithmic IBD from the others (p > 0.06).

### Community analysis

**Figure 3:**
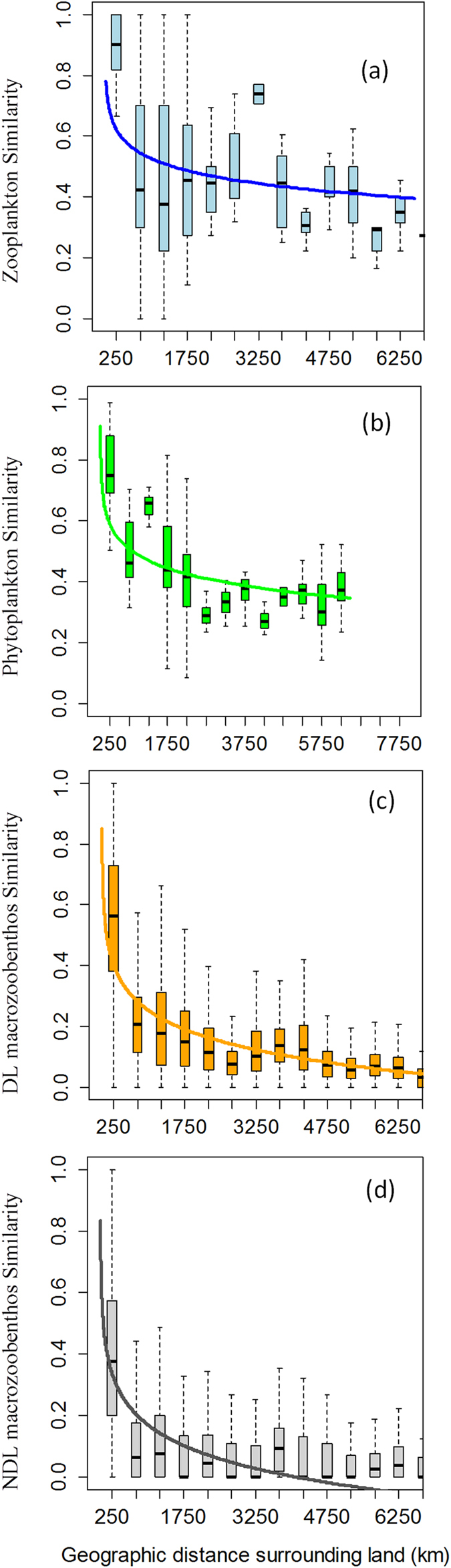
Community similarity *vs*. geographic distance for planktonic and macrozoobenthic groups. Community similarity is fitted with the logarithmic decay model. Boxplots depict data variability at each distance interval. NDL = macrozoobenthos Non-Dispersing Larvae, DL: macrozoobenthos Dispersing Larvae.

Similarity in species composition decreased with the logarithmic distance for all groups (Table 3), showing a strong decay in the first 1000–2000 km and a flat decay beyond that threshold (Fig. 3). For all groups, the Mantel correlation between species similarity and the logarithmic geographic distance was higher than that between species similarity and environment (Table 3). Therefore, halving distances were estimated according to the two fits (logarithmic and exponential), but more reliability was given to the logarithmic value.

Halving distances using both logarithmic and exponential decay as surrogates of dispersal scales were lowest in the NDL macrozoobenthic community (64 km and 1346 km for logarithmic and exponential decay, respectively), followed by DL macrozoobenthic (101 km, 1603 km), phytoplanktonic (826 km, 4051 km), and zooplanktonic (1444 km, 7280 km) communities (Table 4). Break-point detection analysis over geographical distances showed that phytoplanktonic communities were pan-dispersed for threshold distances below ~168 km (Figure S1), while macrozoobenthic community similarities decreased faster up to ~205 km (Figure S1). In general, a strong decay was observed in the first 1000–2000 km and a smooth decay was observed beyond that threshold (Figure S1).

## Discussion

Estimates of dispersal scales derived from population genetic data sorted the biological groups as follows: NDL macrozoobenthic species <DL macrozoobenthic <plankton. This is supported by the ANOVA of the overall data set. These results support why plankton-related studies cover in average a much larger area than do those of macrozoobenthic species (mean sampling range for plankton = 4121.8 ± 2023.8 km and for macrozoobenthos = 1477.3 ± 563.4 km; Figure S2). The specific weight meta-analysis of the data subset also indicated lower dispersal scales for NDL, although no differences were evident between DL and plankton groups. This could be related to the limited amount of data available for this specific analysis (n = 60, out of 138). In particular, the scarcity of IBD studies for phytoplankton (possibly caused by difficulties related to strain isolation and/or monoclonal culture) limited the power of the statistical analysis when compared with other groups.

Marine invertebrates with direct development often display relatively strong genetic population structure in comparison to species with planktonic larval stages^33^,^34^, and strong differences linked to their development mode can be evidenced even within a single cryptic species complex^35^. Nevertheless, factors other than the pelagic duration of larvae, such as the ability to tolerate environmental stress^36^, habitat fragmentation^37^, effective size, and generation time^38^, can explain the genetic structure observed in the populations of these organisms^39^. Even within a development mode and within a cryptic species complex, significant differences in realised connectivity can be observed^40^, suggesting that contingency, such as demographic history, has a potentially strong influence. Furthermore, we estimated the distance between populations using the geographical distance surrounding land without taking into account the hydrodynamics, which can also play an important role in connectivity patterns^41^,^42^,^43^.

At the community level, similarity in species composition decreased with the logarithm of distance for all groups, with a strong decay in the first 1000–2000 km and a flat decay beyond that threshold. For all groups, moreover, the Mantel correlation between species similarity and the logarithm of geographic distance was higher than that with environment, supporting the assumptions of the neutral theory of biodiversity and enabling the inference of a dispersal scale. The dispersal scale ranked the biological groups in support of our hypothesis: NDL macrozoobenthic (64 km) <DL macrozoobenthic (101 km) <phytoplanktic (826 km) <zooplanktonic (1444 km). This was the same as they were ordered for the genetic population analysis. In terms of absolute values, different estimates of dispersal between the two methods are probably due to the use of different similarity indices, sets of localities, and species analysed. The larger halving distance of zooplankton than of phytoplankton might be related to their slightly longer life span and the diel vertical migration of zooplankton, which allows them to use different currents in the water column to their dispersal advantage. This indicates that prevailing habitat (strict pelagic, i.e., phyto- and zooplankton; strict benthic, i.e., NDL macrozoobenthos; or multihabitat, i.e., DL macrozoobenthos) determines the degree of community connectivity.

**Figure 4:**
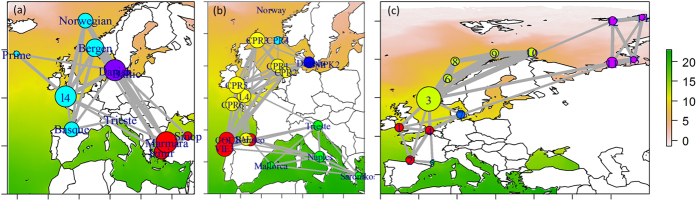
Hierarchical clustering based on the β_sim_ index for (**a**) phytoplanktonic, (**b**) zooplanktonic, and (**c**) macrozoobenthic communities (colours of stations indicate different cluster groups). Size of stations indicates the number of connections (i.e., the similarity between sites). Width of connections indicates the degree of similarity (thicker or thinner for more or less similar, respectively). Connections with similarities below 0.6 were removed. Previous to the analysis, some stations were aggregated according to their proximity for clarity. Network graph maps were generated with *igraph* package (version 1.0.1; URL: http://igraph.org/) and implemented in R language (R Core Team (2015). R: A language and environment for statistical computing. R Foundation for Statistical Computing, Vienna, Austria. URL https://www.R-project.org/).

Results from break-point detection analysis over geographic distances showed that phytoplanktonic communities were “pan-dispersed” for threshold distances lower than ~168 km, while macrozoobenthic communities similarity decreased faster until ~205 km. In general, a strong decay was observed in the first 1000–2000 km and a smooth decay was observed thereafter, which may be associated with a spatial choke point where two main regions (e.g., Mediterranean and Atlantic phytoplanktonic populations, Fig. 4) connect through the Strait of Gibraltar. Shorter distance thresholds identified at logarithmic scales (170–200 km) could be related to individual or propagule dispersal distance because they are of the same order of magnitude as several of the species reported in the analysis of population genetics^44^.

By comparing planktonic and benthic macroinvertebrate assemblages, we show relevant links between community and population genetics. Similarity decreases in both population genetics and community composition with geographic distance, whereby, for communities at least, this is not a resulting pattern of environmental distance. Thus, this appears to be a pattern associated with dispersal limitation for an important number of species and communities. Moreover, both genetic and community analyses show that macrozoobenthic NDL species have lower dispersal scales than do macrozoobenthic DL, and both have lower dispersal scales than do plankton, in agreement with neutral theory expectations. Here, we highlight the similar patterns obtained at both genetic and community levels regardless of the following differences: (i) the use of different similarity indices and sets of localities and species analysed (hence, characterised by different biogeographic histories); (ii) processes such as ecological and genetic drift might act at different time scales; and (iii) limits in the parallels between population genetics and community ecology; for instance, many aspects of the evolutionary process, such as epistasis, pleiotropy, inbreeding, and recombination, have no parallels in community ecology^9^.

The IBD model was well supported in macrozoobenthic groups, but supported by only 3 out of 13 species of phytoplankton tested. To balance the particularities in genetic diversity of individual taxa, such as population similarity reflecting historical rather than contemporary gene flow in some species^4^, a multi-taxon approach is required. Recent developments in sequencing technologies^45^ are now allowing for a much finer resolution of subtle population genetic structures, which will be useful especially for planktonic species.

Beyond the particularities of each species, similarity decreased in population genetics and in species composition consistently with geographic distance for a considerable number of species, where the rate of decline is associated with dispersal limitations. At the genetic level, dispersal scales sorted the groups in the same order as they did at the community level: NDL macrozoobenthos <DL macrozoobenthos <plankton, in agreement with expectations of the neutral theory. Since there are six (i.e., 3 × 2 × 1) possible rankings of three elements, the probability of obtaining this ranking, predicted by dispersal ability at both levels of organisation by chance is 1/6 · 1/6 = 0.028. This statistically significant value provides the first evidence of relevant links between community and population genetics among marine planktonic and benthic macroinvertebrate assemblages. Implications of this finding in terms of how dispersal might affect local species richness and speciation in pelagic *versus* benthic habitats remain to be studied. A practical consequence for biodiversity conservation is that population genetics data from only a few species may help to predict community connectivity patterns, and conversely, β-diversity knowledge may provide useful a priori information to infer single-species connectivity, taking into account differences in dispersal estimates between the two methods.

## Material and Methods

### Genetic population analysis: definitions of biodiversity components and data compilation

We selected three biological marine groups: phytoplankton, zooplankton, and benthic macroinvertebrates (hereinafter called macrozoobenthos). In this study, phytoplankton included diatoms, dinoflagellates, and coccolithophorids; zooplankton included Annelida, Arthropoda (euphausiids, mysids, copepods, and Crustacea), Chaetognata, Cnidaria, Ctenophora, and Nematoda (i.e., all available taxa with a pelagic adult stage), and excluding benthic macrozoobenthic larvae (i.e., meroplankton); and benthic macrozoobenthic taxa included Annelida, Arthropoda (Crustacea), Bryozoa (Cheilostomatida), Chordata (Tunicata), Cnidaria, Echinodermata (spinosulida, ophiurida, camarodonta), Mollusca (Gastropoda), platyhelminthes, and Porifera (dictyoceratida). Macrozoobenthic species were divided into two main groups according to their larval dispersing strategy^46^: (i) dispersing larvae (DL; including both planktotrophic and lecithotrophic larvae characterised by a long (>12 weeks) to short (1 day-12 weeks) pelagic phase); and (ii) nondispersing larvae (NDL; direct developers, brooding, characterised by a larval stage with very low dispersal potential).

We conducted a bibliographic survey of IBD slopes derived from population genetics data to test differences in dispersal scale among marine groups. Inclusion criteria for the selected studies included the availability of (i) either IBD slope or differentiation F_ST_ statistics^19^; (ii) geographic distances among populations or raw genetic data (e.g., haplotypes, molecular markers); and (iii) more than three sites per case study. We used abstracts obtained from the Web of Science (Reuters 2014) (1997–2014), using pairs of combinations of the following keywords as search strings: *genetic, structure, isolation by distance, diversity*, and *population* with *phytoplankton, zooplankton*, and *macrobenthos*. We also included some unpublished data in the analysis. Overall, we analysed 290 papers about plankton (zooplankton and phytoplankton) and 220 papers about macrozoobenthos. Studies were excluded if they included invasive species with recent (i.e., years to decades) invasions to new areas or did not include the geographic locations of sampling points. For those studies (see Table S1 in the supplementary material) that did not include correlation and significance of IBD correlations, we tested the significance of their IBD slopes using reported F_ST_ values and the geographic coordinates of the sampling sites (see next section). We used GENEPOP (http://genepop.curtin.edu.au/) to estimate pairwise F_ST_ values from haplotype frequencies for the few studies that included haplotype frequency matrices.

### Analysis of dispersal scales based on IBD

We compared differences in dispersal scales between macrozoobenthic and planktonic groups. To address this, we searched IBD values for species whose F_ST_ and geographic coordinates were provided. We used *marmap* package in R^47^ to calculate the least-cost distance between sampling points surrounding land and Mantel tests^48^ with a Spearman correlation coefficient and 1000 permutations to assess the significance of the correlation between the sample pairwise genetic differentiation, F_ST_, and geographic distance for each species. We used a four-fold correction factor on the IBD slope for mitochondrial genetic markers rather than the two-fold correction used in Kinlan and Gaines^49^ because the effective size of mitochondrial genomes accounts for the number of females (i.e., a quarter of the number of nuclear genomes assuming a 1:1 sex ratio for diploids). This approach was based on linear regressions of F_ST_ versus distance.

To estimate dispersal distance from IBD slopes at the group level, we applied the method used by Kinlan and Gaines^49^ to our data set based on simulations under a particular stepping-stone model^32^. We used a power function model (dispersal distance = 0.0016 (IBD slope)^−1.0001^) to estimate dispersal distances established in Palumbi^32^. Dispersal estimates represent the equivalent mean dispersal distance required to generate the observed F_ST_/distance slope under the model’s assumptions (stepping-stone model and assumption of a deme size of 1000; see Palumbi^32^).

Because molecular marker choice for determining F_ST_ can affect the outcome of population genetics studies^50^,^51^,^52^,^53^, several precautions were taken when comparing studies using distinct genetic markers. Microsatellites have much higher mutation rates than other markers, in particular compared to allozymes^54^, but mutation rates should not influence IBD parameters under the neutrality hypothesis. However, allozyme polymorphisms are expected to depart from the neutral hypothesis more often than microsatellites, which are noncoding DNA regions, and differences in IBD values could eventually result from this because of the influence of selection on allozyme diversity. Mitochondrial DNA markers represent another case regarding the selective regime (more genetic drift, leading to a lower efficiency of natural selection) and always represent a single locus since the mitochondrial genome does not recombine (high stochasticity). Hence, we tested the effect of the main molecular marker types (allozymes, mitochondrial, and microsatellites) used for each species on the IBD slope for each biological group, including the molecular type as a factor in the statistical test (see below).

Subsequently, we compared differences in dispersal scales between macrozoobenthic and planktonic groups using two approaches. In the first approach, we tested for differences in the mean values of IBD slopes among biological groups using a two-way ANOVA (after normalising data using a logarithmic transformation), one factor for the biological group and the other for the molecular marker type, and a Tukey’s test for pair-wise comparison. To retrieve F_ST_ from studies taking F_ST_/(1 − F_ST_), we performed the corresponding transformation and fit a linear regression taking into account F_ST_ maximum and minimum values, the intercept, and the IBD slope.

The second approach was based on a meta-analysis that integrated the quantitative findings from separate but similar studies and provided a numerical estimate of the overall effect of interest, by taking into account different weights assigned to the different studies to estimate the pooled effect^55^. Studies with smaller standard error and larger sample size were given more weight in the calculation of the pooled effect size. In particular, we conducted a weighted mixed effect model meta-analysis^56^ to test the effect of the predictor variables (marker and group) on the IBD slope by means of the restricted maximum-likelihood estimator. The null hypothesis was that there were no differences in test statistics among groups or markers. The meta-analyses were conducted using the *metafor* package in R^56^. Because this analysis required the variance of the IBD slope, this statistic was estimated from the fit of IBD between the geographic and F_ST_ data, and hence, the number of cases was limited to those where all data was available (i.e., *n* = 60 out of 138). As in the previous approach, two factors were included in the two-way ANOVA (biological group and molecular marker type).

### Definitions of community data sets and compilations

We analysed the species composition of communities of phytoplankton, zooplankton, and soft-bottom macroinvertebrates to quantify the dispersal scale of organisms for each group. The data set detailing information on these species and information on dispersal modes are given below. In all cases, we restricted the data set to marine samples (inner estuarine areas were excluded) and to individuals identified at the species level, removing all taxa identified at higher (e.g., genus) and lower (e.g., subspecies) taxonomic levels to minimise the effect of different taxonomic resolutions used in each study.

We compiled an inventory of phytoplankton from 36 stations (33 were fixed stations and 3 were considered small areas where data were compiled from different studies). Stations included the Atlantic Margin and North Sea^57^, the southeastern Bay of Biscay^58^, the Kattegat strait, the southwestern Baltic Sea^59^, and Sinop Bay (see references in Table S2) and the Gulf of Trieste (North Adriatic^60^, the Sea of Marmara (see references in Table S2), and Izmir Bay (eastern Aegean Sea, see references in Table S2) from the Mediterranean Sea. We restricted the data set to three phytoplankton groups (diatoms, dinoflagellates, and coccolithophorids) and overall 555 species were identified.

We compiled an inventory of zooplankton (restricted to copepods as representative of zooplankton communities because they are the most diverse and are commonly identified at the species level) using data from 27 fixed stations of copepod community data from the Atlantic Margin, the North Sea, the Norwegian Sea (NMFS-COPEPOD global plankton database^61^, the Bay of Biscay^62^,^63^, the Kattegat strait, and the southwestern Baltic Sea (unpublished data) from the European Seas (Table S3) and the Gulf of Trieste^64^, the Gulf of Naples^65^,^66^, Saronikos Gulf, and southwest of Mallorca island^67^,^68^ from the Mediterranean Sea. We checked species names using WoRMS^69^ to avoid synonyms and duplicates. The overall data set resulted in 179 species of copepod.

We compiled an inventory of soft-bottom macrozoobenthic species from three data sources: (i) the pan-European MacroBen database^70^ (available at EMODnet Biology portal (http://bio.emodnet.eu/portal), see complete reference list in Table S4), covering the Irish Sea, the North Sea, the Norwegian Sea, Barents Sea, and the Gulf of Lion (eastern Mediterranean), including 1814 sampling locations that were spatially (~10 by 10 km) and temporally aggregated into 305 stations. (ii) The Basque water quality network (19 fixed coastal stations were sampled during 2003–2008 and were spatially and temporally aggregated into 17 stations; see Borja *et al*.^71^) covering the Basque coast (the southeastern Bay of Biscay). (iii) A Danish data set covering the Kattegat strait and the southeastern Baltic Sea^72^ (1415 sampling locations were sampled during 1990–2013 and were spatially and temporally aggregated into 271 stations) (http://www.dmu.dk/en/water/marinemonitoring/mads/plankton/). Macrozoobenthic taxa were sampled with a grab within 0.04 to 1 m^2^ of the surface of soft-bottom sediment, where most occupied 0.1 m^2^. We restricted stations sampled between 0 and 450 m depth (all stations without depth information were removed), between 1990 and 2013 (to reduce heterogeneity in temporal changes), and at a minimum of 10 km between samples (those closer were aggregated). With these filtering criteria, the overall data set comprised 593 stations and 2276 species. The macrozoobenthic group was divided into two sub-groups according to dispersal types of 2193 species: 1345 species belonged to the dispersing larvae group and 848 belonged to the nondispersing larvae group.

### Environmental data for community analysis

We obtained environmental data from the records of each biological station and took an averaged of those points with multiple samplings; in the case of unavailable data, we sourced Bio-Oracle^73^ and NOAA ETOPO1^74^. For phytoplankton, we analysed seven environmental variables: sea surface temperature (SST), surface salinity, dissolved oxygen, Secchi depth, ammonium-nitrogen (NH_4_-N), total nitrogen, and total phosphorus. For zooplankton, we analysed six environmental variables: depth, SST, salinity, dissolved oxygen, chlorophyll-*a*, and the diffuse attenuation coefficient. For macroinvertebrates, we analysed seven environmental variables: depth, SST, surface salinity, dissolved oxygen, nitrate ([NO_3_] and [NO_3_ + NO_2_],), phosphate (ortho-phosphate concentration [HPO_4_^−2^]), and a diffuse attenuation coefficient at 490 nm (m^−1^).

### Community species similarity

We computed pairwise species similarity among sites for each group separately (macrozoobenthic, phytoplankton, and zooplankton). We used a narrow sense dissimilarity index that focused on compositional differences independent of species richness gradients^75^: β_sim_^76^. This expresses the proportion of shared species with respect to the minimum number of species of the two sites as


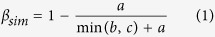


where *a* is the number of species shared between the two sites and *b* and *c* are the total number of species that occur in sites 1 and 2, respectively. The aim of this index is to prevent problems related to the number of species at each site, which differs mainly because of different sampling efforts. For macroinvertebrates, some sites were sampled only once, while others were sampled 2 to 30 times. For phytoplankton, sites were sampled between 19 and 316 times and for zooplankton, sites were sampled between 12 and 787 times.

The geographic distance matrix was defined as the minimum path distance (km) between two pairs of sites across the sea, circumventing the terrestrial zone; this was computed using *unicor* software^77^ and *marmap* package in R^47^. *Unicor* applies Dijkstra’s shortest path algorithm to individual-based simulations. We assigned a resistance value of 1 to all marine pixels; thus, the distance matrix is given in distance (km) units. Because of computational limits, the resistance layer (i.e., binary map marine/land) had a spatial resolution of 10 km for macroinvertebrates, 3.3 km for phytoplankton, and 14 km for zooplankton.

We performed Mantel correlation tests and partial Mantel tests^48^ between species similarity, geographic distance, and environmental distance for causal modelling and inferring marine connectivity. Because distance decay may also result from the relationship between species composition and environmental niche factors^14^,^27^,^78^, firstly, we performed partial Mantel tests to determine the relative contribution of geographic and environmental distances in accounting for species composition similarity. Pairwise environmental distances were computed using the Euclidean distance. To test the correlation between species similarity and environmental distance, we first selected the best subset of environmental variables, such that the Euclidean distance of scaled environmental variables would have a maximum correlation with community dissimilarities; this was done using the *vegan* package^79^ implemented in R 2.13.1 language (R Development Core Team, 2011). We then compared the possible *2*^*p*^−*1* models, where p is the number of environmental variables for each community group. Subsequently, we undertook a partial Mantel test to determine the relative contribution of environmental (after model selection) and geographic distances in accounting for species variation.

We inferred dispersal scales and compared among species groups by estimating halving distances as a measure of the distance-decay rate (i.e., species similarity decay with (geographic) distance^11^) using two approaches. (i) The logarithmic decay model, expressed as *1* − *S* = *c* ln(*d*), where *S* is similarity at distance *d* and *c* is the rate of distance decay, assuming *S* = 1 when *d* = 0; the corresponding halving distance, at which the similarity is half its initial value is *d*_*H*_ = *e*^0.5/c^. (ii) The exponential decay model expressed as *S* = *S*_*0*_*e*^−cd^, where *S*_*0*_ is the initial similarity^80^ and the corresponding halving distance is *d*_*HD*_ = *−*(ln(0.5))/*c*. Additionally, we used the fit of distance decay curves with local polynomial regression functions^81^ to identify thresholds in those curves using breakpoint detection from generalised linear models with piecewise linear relationships^82^.

We performed network graphs that show spatial patterns of community groups and the degree of connectivity among them with *igraph* package in R language^83^. First, we aggregated the number of stations (593 for macrobenthos and 36 for phytoplankton) into limited, representative areas according to their proximity (14 groups for macrobenthos and 11 for phytoplankton). Second, we regrouped species matrices using hierarchical clustering into groups according to the β_sim_^76^. Subsequently, we generated network graphs specifying the following parameters: vertices (i.e., sites) denoted locations where size was proportional to the number of connections (i.e., the similarity between sites), colour represented clustered groups, edges (i.e., connections) had widths that were proportional to the degree of dissimilarity (thicker and thinner edges represent more or less similar, respectively). We removed connections with dissimilarities larger than 0.6 for clarity.

## Additional Information

**How to cite this article**: Chust, G. *et al*. Dispersal similarly shapes both population genetics and community patterns in the marine realm. *Sci. Rep*. **6**, 28730; doi: 10.1038/srep28730 (2016).

## Supplementary Material

Supplementary Information

Supplementary Table S1

## Figures and Tables

**Figure 1:**
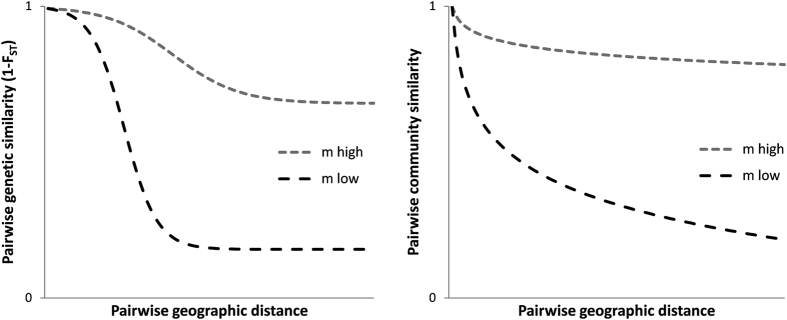
(**a**) Decrease of genetic similarity (1 − F_ST_) with geographic distance under a stepping-stone model (isolation-by-distance plot). *m* is the migration rate among subpopulations in a metapopulation, modified from Selkoe and Toonen^20^. (**b**) According to the neutral model of biodiversity, species cross-site similarity is predicted to decline logarithmically with increasing geographic distance as a function of migration rates. *m* is the migration among subcommunities in a metacommunity.

**Table 1:** Values for the isolation-by-distance (IBD) slope and dispersal scale (km) for each group.

Group	Mean of IBD slopes	Dispersal scale (km)	N_sig_	N_total_
Macroinvertebrates			66	98
Macro-NDL	0.005168	0.31	15	17
Macro-DL	0.000835	1.92	51	81
Plankton			9	35
Phytoplankton	0.000082	19.53	3	13
Zooplankton	0.000018	88.99	6	22

N_sig_ = Number of species with significant IBD slopes. N_total_ = Total number of species analysed. NDL = Non-dispersal larvae. DL = Dispersal larvae. A correction factor was applied to the IBD slope for mitochondrial cases.

**Table 2:** Tukey’s test for the log IBD values for pairwise comparison among biological groups and among molecular marker types.

		Difference	p-value
Biological group	NDL-DL	2.421	**0.0038**
	Phytoplankton-DL	−0.216	0.9968
	Zooplankton-DL	−1.773	**0.0258**
	Phytoplankton-NDL	2.637	0.1165
	Zooplankton-NDL	−4.194	**<0.0001**
	Zooplankton-Phytoplankton	−1.556	0.5169
Molecular marker type	Microsatellites-Allozyme	−0.585	0.5835
	Mitochondrial-Allozyme	−1.562	**0.0187**
	Mitochondrial-Microsatellites	−0.977	0.2577

NDL = macrozoobenthos Non-Dispersing Larvae, DL: macrozoobenthos Dispersing Larvae.

**Table 3:** Mantel (r_M_) and partial Mantel tests between species similarity and geographic distance surrounding land and environmental determinants for each taxonomic group and for each approach (logarithmic decay where *S* declines with ln of distance, and exponential decay expressed as *S* = *S*_*0*_*e*^*−*cd^).

Model	Variable	Phytoplankton		Zooplankton		Macrozoobenthos		Macrozoobenthos DL		Macrozoobenthos NDL	
		r_M_	p-value	r_M_	p-value	r_M_	p-value	r_M_	p-value	r_M_	p-value
Logarithmic decay	Geographic distance	0.77	0.0001	0.63	0.0001	0.69	0.0001	0.69	0.0001	0.56	0.0001
	Environment	0.49	0.0001	0.36	0.0001	0.49	0.0001	0.50	0.0001	0.37	0.0001
	Geographic distance, partialling out environment	0.72	0.0001	0.63	0.0001	0.62	0.0001	0.61	0.0001	0.49	0.0001
Exponential decay	Geographic distance	0.64	0.0001	0.39	0.0001	0.35	0.0001	0.34	0.0001	0.24	0.0001
	Environment	0.42	0.0001	0.21	0.0001	0.32	0.0001	0.31	0.0001	0.22	0.0001
	Geographic distance, partialling out environment	0.57	0.0001	0.39	0.0001	0.29	0.0001	0.28	0.0001	0.19	0.0001

NDL = macrozoobenthos Non-Dispersing Larvae, DL: macrozoobenthos Dispersing Larvae.

**Table 4:** Halving distances from logarithmic and exponential decay models for each species group.

	Logarithmic decay *1−S* = c ln(*d*)		Exponential decay *S* = *S*_*0*_*e*^−cd^		
	Slope (c)	Halving distance (km) *d*_*HD*_ = *e*(0.5*/c*)	Slope (c)	S_0_	Halving distance (km) *d*_*HD*_ = *−*(ln(0.5))*/c*
Macrozoobenthos	0.1111	90.1	4.334e–04	0.25	1599.3
-NDL	0.1202	64.1	5.150e–04	0.06	1345.9
-DL	0.1084	100.7	4.325e–04	0.25	1602.6
Phytoplankton	0.0744	826.1	1.711e–04	0.65	4051.1
Zooplankton	0.0687	1444.3	9.520e–05	0.54	7280.9
