# Supplementary material for: Dispersal similarly shapes both population genetics and community patterns in the marine realm
Source: Sci Rep. 2016 Jun 27;6:28730. doi: 10.1038/srep28730 (PMC4921837; doi:10.1038/srep28730)
Supplement: Supplementary Information [file srep28730-s1.pdf]

Dispersal similarly shapes both population genetics and community patterns in the marine realm

Author list: Guillem Chust, Ernesto Villarino, Anne Chenuil, Xabier Irigoien, Nihayet Bizsel, Antonio Bode1, Cecilie Broms, Simon Claus, María L. Fernández de Puelles, Serena Fonda-Umani, Galice Hoarau, Maria G. Mazzocchi, Patricija Mozetič, Leen Vandepitte, Helena Veríssimo, Soultana Zervoudaki, Angel Borja

Supplementary Information

Tables S1 to S3

Figures S1 to S2

Supporting Online Material

Table S1. Genetic population data and sources.

Table S2. Data sources of the phytoplanktonic community.

Region	Sites	N. samplings per site analysed	Period	Sources
Mediterranean	Gulf of Trieste (N Adriatic)	120 (monthly at 3-4 depths)	2005-2007	Mozetič et al. 2012
Mediterranean	Izmir	99 (from 11 cited studies)	1978-2013	See references below
Mediterranean	Marmara	140 (from 10 cited studies)	1974-2010	See references below
Black Sea	Sinop Bay	At least 23 samplings (from 9 cited studies)	1995-2004	See references below
Atlantic Margin	Bergen (North Sea)	20	2000	Sal et al., 2013
Atlantic Margin	"L4" station	20	2001	Sal et al., 2013
Atlantic Margin	Norwegian	19	1997	Sal et al., 2013
Atlantic Margin	Prime	20	1996	Sal et al., 2013
Bay of Biscay	14 sites in Basque coast	15-22 depending on site	2003-2008	Borja et al., 2009
Kattegat and Baltic Sea	14 sites	34-316	1979-2012	Carstensen et al. 2007
Total	***36***			

References

Borja, A., J. Bald, J. Franco, J. Larreta, I. Muxika, M. Revilla, J. G. Rodríguez, O. Solaun, A. Uriarte, V. Valencia, 2009. Using multiple ecosystem components, in assessing ecological status in Spanish (Basque Country) Atlantic marine waters. Marine Pollution Bulletin, 59: 54-64.

Carstensen, J., Henriksen, P. & Heiskanen, A.S. (2007) Summer algal blooms in shallow estuaries: Definition, mechanisms, and link to eutrophication. Limnology and Oceanography, 52, 370-384.

Mozetič, P., Francé, J., Kogovšek, T., Talaber, I., Malej, A. (2012) Plankton trends and community changes in a coastal sea (northern Adriatic): Bottom-up vs. top-down control in relation to environmental drivers. Estuarine, Coastal and Shelf Science, 115, 138-148.

Sal, S., López-Urrutia, Á., Irigoien, X., Harbour, D.S. & Harris, R.P. (2013) Marine microplankton diversity database. Ecology, 94, 1658.

References for data from Izmir Bay:

Bizsel N and Nezan E (2007). The new phytoplankton records from Turkey. CIESM, Rapp. Comm. int. Mer Médit., 38, pp:350.

Bizsel, N. 2000. Iron forms and their role on Phytoplankton ecology. The project supported by the scientific and technical research council of Turkey.

Çolak F S and Koray T (2007). Influence of the sewage treatment plant on Diatom (Bacillariophyceae) species composition in Izmir Bay. E.U. Journal of Fisheries & Aquatic Sciences, 24:1-2, 11-18.

Çolak, F. 2000. İzmir Körfezi Mikroplankton'unun Kalitatif ve Kantitatif Dağılımı. Ege Üniversitesi, Su Ürünleri Fakültesi Master Tezi.

Göçer S. 2001. Fitoplanktonik Toksinlerin Gıda Zincirindeki Denizel Canlılarda Tayini Üzerine Bir Araştırma. Doktora Tezi.

Inanan, B. E. 2007. İzmir Körfezi Fitoplankton Kompozisyonu ve Kompozisyonu Etkileyen Parametreler. Dokuz Eylül Üniversitesi, Deniz Bilimleri Enstitüsü Master Tezi.

Koray T. 1995. Phytoplankton species succession, diversity and nutrients in neritic waters of the Aegean Sea (Bay of Izmir). Turk J. Bot. 19:531-544.

Koray, T. 1985. İzmir Körfezi'nin Mikroplanktonunda Meydana Gelen Değişimlerde Ortam Faktörlerinin Rolü. Ege Üniversitesi, Biyoloji Bölümü, Hidrobiyoloji Ana Bilim Dalı Doktora Tezi.

Metin G. 1995. İzmir Körfezi'ndeki Fitoplankton'un Pigment Maddeleri ve Partikül Organik Karbon İlişkilerine Bağlı Olarak Aylık Kantitatif Değişimleri Üzerine Bir Araştırma. Doktora Tezi.

Sabancı, Colak F and Koray T. 2005.İzmir Körfezi'nde 1998-2001 Yılları Arasında Fitoplanktonik Tür Çeşitliliği Değişimi. Ege Su Ürünleri Dergisi, Vol:22 (3-4): 273-280.

Tümer, T. 2012. Daily Phytoplankton Distribution in Inciraltı, Izmir Bay. Dokuz Eylül University, Institute of Marine Sciences and Technology, Master Thesis.

References for data from the Black Sea:

Bat, L. et al.2011. Biological Diversity of the Turkish Black Sea Coast. Turkish Journal of Fisheries and Aquatic Sciences 11: 683-692. DOI: 10.4194/1303-2712-v11_4_04.

Bircan R. 2005. Karadeniz'in Sinop bölgesinin alt besin tabakalarının dinamik ve zaman serileri. TUBITAK Projesi (Proje Numarası:199Y121).

Büyükhatipoğlu S. 2002. Orta Karadeniz'in Sinop Burnu Bölgesinin Biyokimyasal Dönüşüm Çalışmaları. TUBITAK Projesi (Proje Numarası:619/G).

Feyzioğlu A. M. 2010. Doğu Karadeniz Kıyısal Ekosisteminde Fitoplankton Biyomass ve Pigment Kompozisyonunun HPLC Yöntemi ile Belirlenmesi. TUBITAK Projesi (Proje Numarası:108Y241).

Gomez F and Boicenco L. 2004. An annotated checklist of dinoflagellates in the Black Sea. Hydrobiologia 517:43-59.

Moncheva S., Petrova-Karadjova V., Palasov A. Harmful algae blooms along the Bulgarian Black Sea coast and possible patterns of fish and zoobenthic mortalities. In: Harmful Marine Algal Blooms / P Lassus, G.Arzul, E.Denn, P. Gentien [eds], Lavoisier Publ. Inc., 1995, P. 193-198.E.Denn, P. Gentien [eds], Lavoisier Publ. Inc., 1995, P. 193-198.

Seyhan K. 2005. Doğu Karadeniz'de Alt Besin Tabakalarının Dinamiği. TUBITAK Projesi (Proje Numarası:100Y072).

Turkoglu M and Koray T 2004. Algal Blooms in Surface Waters of the Sinop Bay in the Black Sea, Turkey. Pakistan J. of Biological Sciences 7: 1577-1585.

Turkoglu M and Koray T. 2002. Phytoplankton Species' Succession and Nutrients in the Southern Black sea (Bay of Sinop). Turkish Journal of Botany. Vol:26, 235-252.

References for data from the Sea of Marmara:

Altug G. 2007. Kuzey Ege ve Güney Marmara Denizi Biyolojik Çeşitliliğinin Fiziksel Kimyasal ve Biyolojik Verilerle Değerlendirilmesi. TUBITAK Projesi (Proje Numarası:105Y039).

Balkıs N. 2003. Seasonal variations in the phytoplankton and nutrient dynamics in the neritic water of Buyukcekmece Bay, Sea of Marmara. J. of Plankton Research, Vol:25, 7, 703-717.

Balkıs N. 2004. List of Phytoplankton of the Sea of Marmara. J. Black Sea/Mediterranean Environment. Vol:10, 123-141.

Balkıs N. et al. 2004. Summer Phytoplankton Composition in the Neritic Waters of the Sea of Marmara. Pak. J. Bot., 36(1): 115-126.

Deniz N and Taş S. 2009. Seasonal variations in the phytoplankton community in the north-eastern Sea of Marmara and a species list. Journal of the Marine Biological Association of the United Kingdom, 2009, 89: 269–276.

Polat C 2000. Marmara Denizi ve Karadeniz Ekolojik Etkileşiminin Zaman Serisi Verileri ile Araştırılması. TUBITAK Projesi (Proje Numarası: YDABCAG-578/G (197Y024)).

Polat Colpan (2011). Deniz Ortamında Musilaj/Mukus Oluşumunu Denetleyen Faktörlerin Laboratuvar Koşullarında İzlenmesi. TUBITAK Projesi (108Y083).

Tas S. 2011. Haliç'te Zararlı Mikroalgler ve Çevresel Şartların İncelenmesi. TUBITAK Proje Raporu (Proje Numarası:109Y046).

Tufekci, V., Balkis N., Beken P C., Ediger D., Mantıkcı M. 2010.Phytoplankton composition and environmental conditions of a mucilage event in the Sea of Marmara. Turk. J. of Biology, 34:199-210.

Uysal Z. 1996. A net-plankton study in the bosphorus junction of the Sea of Marmara. Turkish Journal of Botany. 20: 321-327.

Table S3. Data sources for the zooplanktonic community.

Region	Sites	N. samplings per site analysed or Frequency	period	Species richness	Sources and references
Kattegat and Baltic Sea	9 sites	3-5 samplings per year over 33 years	1980-2012	26	Unpublished data
South-eastern Bay of Biscay (Bermeo)	1	Monthly over a year	April 2003 to April 2004	13	Albaina and Irigoien 2007
Southern Bay of Biscay (Galicia, Asturias, Santander)	3	Coruña: 144<br>Vigo: 134<br>Santander: 121	Coruña: 1994-2006<br>Vigo: 1994-2006<br>Santander: 1991-2001	Coruña: 52<br>Vigo: 38<br>Santander: 34	Bode et al. 2012
L4 off Plymouth in the Western Channel	1	Weekly over 20 years	1988–2007	24	Eloire et al. 2010
CPR (NW Atlantic)	6	Irregular over 5 years (28 - 49 samplings)	1995 to 1999	7-12	NMFS-COPEPOD[1] global plankton data base
Mediterranean (Gulf of Trieste)	1	Monthly over 7 years	1998 - 2005	33	Conversi et al, 2009
Mediterranean (Mallorca)	1	Monthly over 2 years	2003-2004	50	Fernandez de Puelles et al., 2007, 2009
Mediterranean (Gulf of Naples)	1	787 over 19 years	1995-2013	130	Mazzocchi et al. (2011, 2012)
Mediterranean (Saronikos)	1	14 samplings over 3 years	2005-2007	45	Unpublished data
Norway	1	44 over 4 years	2010-2014	22	Unpublished data
Total	*27*			*179*	

[1] http://www.st.nmfs.noaa.gov/copepod/data/sahfosatl/index.html

References

Albaina, A. & Irigoien, X. (2007) Zooplankton communities and oceanographic structures in a high-resolution grid in the south-eastern corner of the Bay of Biscay. Estuarine, Coastal and Shelf Science, 75, 433-446.

Bode, A., Alvarez-Ossorio, M.T., Miranda, A., López-Urrutia, A. & Valdés, L. (2012) Comparing copepod time-series in the north of Spain: Spatial autocorrelation of community composition. Progress in Oceanography, 97–100, 108-119.

Conversi A, Peluso T, Fonda-Umani S (2009) The Gulf of Trieste: a changing ecosystem. J Geophys Res, Oceans 114 C03S90. Doi:10.1029/2008JC004763.

Eloire, D., Somerfield, P.J., Conway, D.V.P., Halsband-Lenk, C., Harris, R. & Bonnet, D. (2010) Temporal variability and community composition of zooplankton at station L4 in the Western Channel: 20 years of sampling. Journal of Plankton Research, 32, 657-679.

Fernández de Puelles, M.L., Alemany, F., Jansá, J., 2007. Zooplankton time-series in the Balearic Sea (Western Mediterranean): variability during the decade 1994–2003. Prog. Oceanogr. 74, 329–354.

Fernández de Puelles, M.L., López-Urrutia, A., Morillas, A., Molinero, J.C., 2009. Seasonal variability of copepod abundance in the Balearic region as a indicator of basin scale hydrological changes. Hydrobiologia 617, 3–16.

Mazzocchi, M.G., Dubroca, L., Garcia-Comas, C., Di Capua, I., Ribera d’Alcalà, M., 2012. Stability and resilience in coastal copepod assemblages: The case of the Mediterranean long-term ecological research at stn MC (LTER-MC). Progress in Oceanography, 97-100, 135-151.

Mazzocchi, M.G., Licandro, P., Dubroca, L., Di Capua, I., Saggiomo, V., 2011. Zooplankton associations in a Mediterranean long-term time-series. Journal of Plankton Research, 33, 1163-1181.

Table S4. Data sources for references for the macrozoobenthic community.

Koukouras A., 2000: Northern Aegean dataset. Aristotelian University of Thessaloniki Department of Zoology and Zoological Museum, School of Biology, Greece. Metadata available at http://www.vliz.be/nl/imis?module=dataset&dasid=630

The Norwegian Oil Industry Association, 2001: Offshore reference stations, North/Norwegian sea. The Norwegian Oil Industry Association (OLF), Akvaplan-niva and Det Norske Veritas, Norway. Metadata available at http://www.vliz.be/nl/imis?module=dataset&dasid=527

Dahle S., R. Palerud, N. Anisimova, 1992: Benthic fauna around Franz Josef Land. Akvaplan-niva, Norway. Metadata available at http://www.vliz.be/nl/imis?module=dataset&dasid=11

Mackie, A.S.Y., P.G. Oliver, E.I.S. Rees, 1991: Biomôr 1 dataset. Benthic data from the Southern Irish Sea from 1989-1991. National Museum and galleries of Wales, Cardiff, UK. Metadata available at http://www.vliz.be/nl/imis?module=dataset&dasid=1600

The Norwegian Oil Industry Association, 2000: Offshore reference stations, Finnmark. The Norwegian Oil Industry Association (OLF), Akvaplan-niva and Det Norske Veritas, Norway. Metadata available at http://www.vliz.be/nl/imis?module=dataset&dasid=527

Dahle S., S. Cochrane, S. Denisenko., 1992: Benthic fauna around Pechora Sea. Akvaplan-niva, Norway. Metadata available at http://www.vliz.be/nl/imis?module=dataset&dasid=461

Bachelet, G., 2004: Benthos Gironde Estuary. Université Bordeaux 1, Station Marine d'Arcachon, Laboratoire d'Océanographie Biologique, France. Metadata available at http://www.vliz.be/nl/imis?module=dataset&dasid=597

Grémare, A., J.M. Amouroux, , C. Labrune, 1998: Redit dataset. Observatoire Océanologique de Banyuls-Sur-Mer; Laboratoire d'Océanographie Biologique, France. Metadata available at http://www.vliz.be/nl/imis?module=dataset&dasid=213

Degraer, S., G. Van Hoey, W. Willems, J. Speybroeck & M. Vincx, 2003: MacroDat Belgium. Macrobenthic data from the Belgian part of the North Sea from 1976 onwards. Ghent University, Biology Department, Marine Biology Section, Belgium. Metadata available at http://www.vliz.be/nl/imis?module=dataset&dasid=633

Sarda R., 1997: Soft Bottom Communities of the Bay of Blanes. Centre d’Estudis Avançats de Blanes (CEAB) Consejo Superior de Investigaciones Científica (CSIC), Spain. Metadata available at http://www.vliz.be/nl/imis?module=dataset&dasid=603

The Norwegian Oil Industry Association, 2002: Offshore reference stations, Norwegian/Barents Sea. The Norwegian Oil Industry Association (OLF), Akvaplan-niva and Det Norske Veritas, Norway. Metadata available at http://www.vliz.be/nl/imis?module=dataset&dasid=527

Janas U., 2002: Gulf of Gdansk dataset. University of Gdansk; Institute of Oceanography; Department of Marine Biology and Ecology, Poland. Metadata available at http://www.vliz.be/nl/imis?module=dataset&dasid=612

Petrov A. & N. Revkov, 1986: MegFeod-Black Sea dataset IBSS, Sevastopol. Institute of Biology of the Southern Seas (IBSS), Ukraine. Metadata available at http://www.vliz.be/nl/imis?module=dataset&dasid=626

Zenetos A., 1987: Aegean Sea coastal benthic communities, Geras gulf (Mytilini), Chemical pollution (tannery effluents). Hellenic Centre for Marine Research, Greece. Metadata available at http://www.vliz.be/nl/imis?module=dataset&dasid=622

Zenetos A., 1990: Seasonal zoobenthos, Saronikos. Hellenic Centre for Marine Research, Greece. Metadata available at http://www.vliz.be/nl/imis?module=dataset&dasid=289

Petrov A. & N. Revkov, 1993: Strelbay-Black Sea” dataset – IBSS, Sevastopol. Institute of Biology of the Southern Seas (IBSS), Ukraine. Metadata available at http://www.vliz.be/nl/imis?module=dataset&dasid=627

Petrov A. & Revkov N., 1986: Jalta-Black Sea dataset – IBSS, Sevastopol. Institute of Biology of the Southern Seas (IBSS), Ukraine. Metadata available at http://www.vliz.be/nl/imis?module=dataset&dasid=628

Zenetos A., 1991: Kalamitsi dataset. Hellenic Centre for Marine Research, Greece. Metadata available at http://www.vliz.be/nl/imis?module=dataset&dasid=623

Zenetos A. & C. Bogdanos, 1992: Kerkyra dataset. Hellenic Centre for Marine Research, Greece. Metadata available at http://www.vliz.be/nl/imis?module=dataset&dasid=624

Zenetos A., 1986: Zoobenthos Kyklades, Aegean Sea dataset. Hellenic Centre for Marine Research, Greece. Metadata available at http://www.vliz.be/nl/imis?module=dataset&dasid=625

Nevrova H., Petrov A. & Revkov N., 1996: Laspibay-Black Sea dataset IBSS, Sevastopol. Institute of Biology of the Southern Seas (IBSS), Ukraine. Metadata available at http://www.vliz.be/nl/imis?module=dataset&dasid=629

Dounas C., 1996: Environmental impact assessment of oil pollution accident in Gialova lagoon and Navarino Bay. Hellenic Centre for Marine Research, Greece. Metadata available at http://www.vliz.be/nl/imis?module=dataset&dasid=272

Figures SI

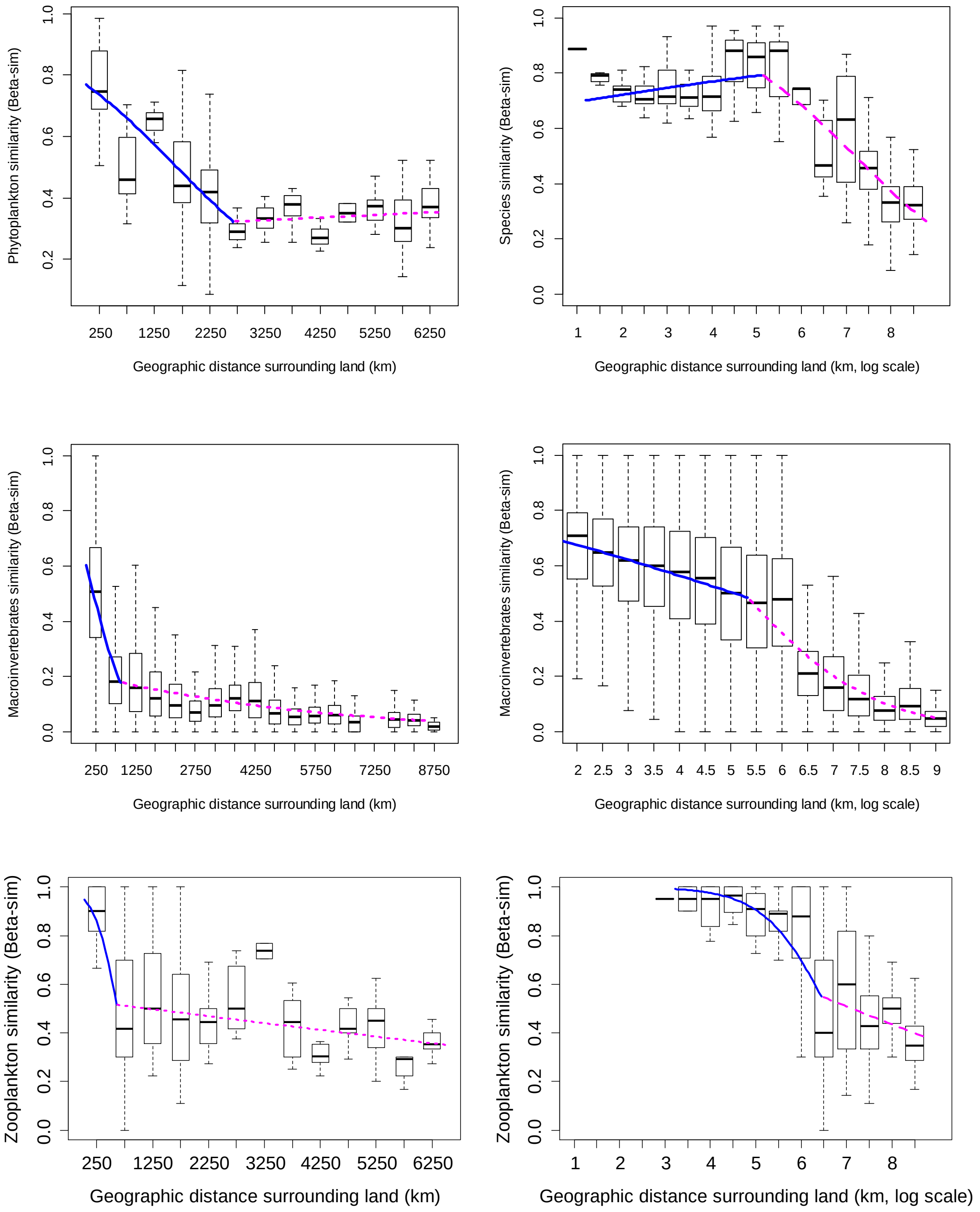

Figure S 1. Detection of breakpoint from generalized linear models. Phytoplankton: breakpoints at 168 km and 2674 km; macrozoobenthos : breakpoints at 205 km and 869 km; zooplankton: breakpoints at 608 km and 629 km. Figures on the right-hand-side are in log scale.

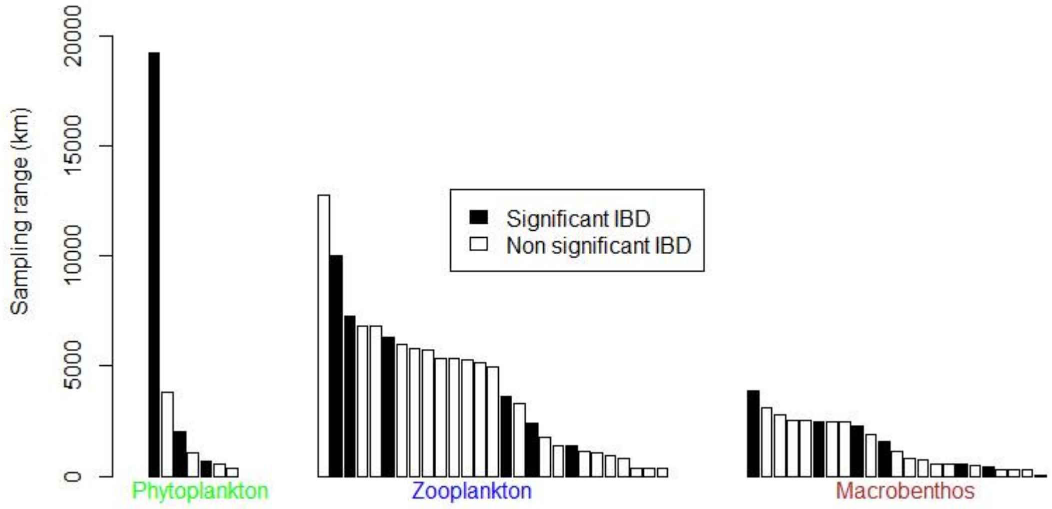

Figure S 2: Sampling range (km) for each biological group and according to significant and non-significant IBD slopes. Each bar represents a single species and species are ordered by a decreasing dispersal scale on the *x* axis.
